# How commerce influences health and oral health

**DOI:** 10.1038/s41415-026-9791-7

**Published:** 2026-11-13

**Authors:** Ivor G. Chestnutt, Anwen L. Cope

**Affiliations:** Dental Public Health, Cardiff University School of Dentistry, Cardiff, Wales, United Kingdom

## Abstract

There is increasing recognition that commercial factors can influence health and oral health both negatively and positively. This narrative review summarises the tactics used to market sugar, tobacco and alcohol, products that can be harmful to oral health. This article considers options open to governments and official agencies to combat the adverse influence of commerce on oral health. It also examines the role of the dental care system itself as a commercial determinant. Finally, it also reviews how commercial determinants can positively influence oral health.

Dental professionals need to be aware of the influence of commercial actors on oral health. Such knowledge can enable them to act as advocates for health and oral health improvement and to engage meaningfully in wider societal debates.

## Key points

- To make significant improvements in oral health and address inequalities, steps need to be taken to control the manufacture and marketing of products with the potential to induce oral disease.
- It is important that dental professionals understand how commercial actors work to promote their products and the impact of such actions.
- Dental professionals have a duty as part of the wider healthcare team to lobby government, local authorities and elected representatives to act in the interests of the population rather than the interests of industry.

## Introduction

‘Money, money, money…it's a rich man's world', according to some of the 20^th^ century's most popular musicians.^1^ Money they say makes the world go around^2^ and what's more, ‘the love of money is the root of all evil'.^3^ There are endless cultural, literary, religious and educational references that could be quoted as an introduction to a discussion on commerce. However, in the context of both general and oral health, one factor is undoubtedly true about money – it has a profound influence.

The impact of social and economic circumstances on wellbeing via lifestyles and life circumstances is thoroughly documented and much discussed.^4^^,^^5^^,^^6^ Increasingly, attention is turning to the role of industry and commerce in influencing health. Understanding and mitigating the negative influences of these commercial forces is a key aspect of population-level health improvement and is a topic on which many health researchers are currently focused.^7^

This paper aims to provide a narrative overview of how money and commerce affect health and oral health, both for the better and for the worse. As a review of publicly available papers and information, formal research ethics review was not necessary for this work.

## The commercial determinants of health

Commercial determinants of health are the private sector activities that affect people's health, directly or indirectly, positively or negatively.^8^

The World Health Organization suggests that in Europe, four major commercial products (alcohol, tobacco, processed food and beverages, fossil fuels) and commercial practices such as exposure to occupation-related carcinogens, asthmagens, and injuries, cause nearly one-quarter (24.5%) of all deaths: 2.7 million annually or about 7,400 daily.^9^ An understanding of how commercial actors work to safeguard their interests and the subsequent influence on health, is a key element of public health practice.

However, not all impacts of commercial companies are negative influences. The many technological advances seen in dentistry and oral health over the past half century, such as advances in dental materials, equipment, or in preventive dental products, such as toothpaste, have relied on industry and the commercial sector.

## Upstream versus downstream approaches to oral health improvement

**Fig. 1:**
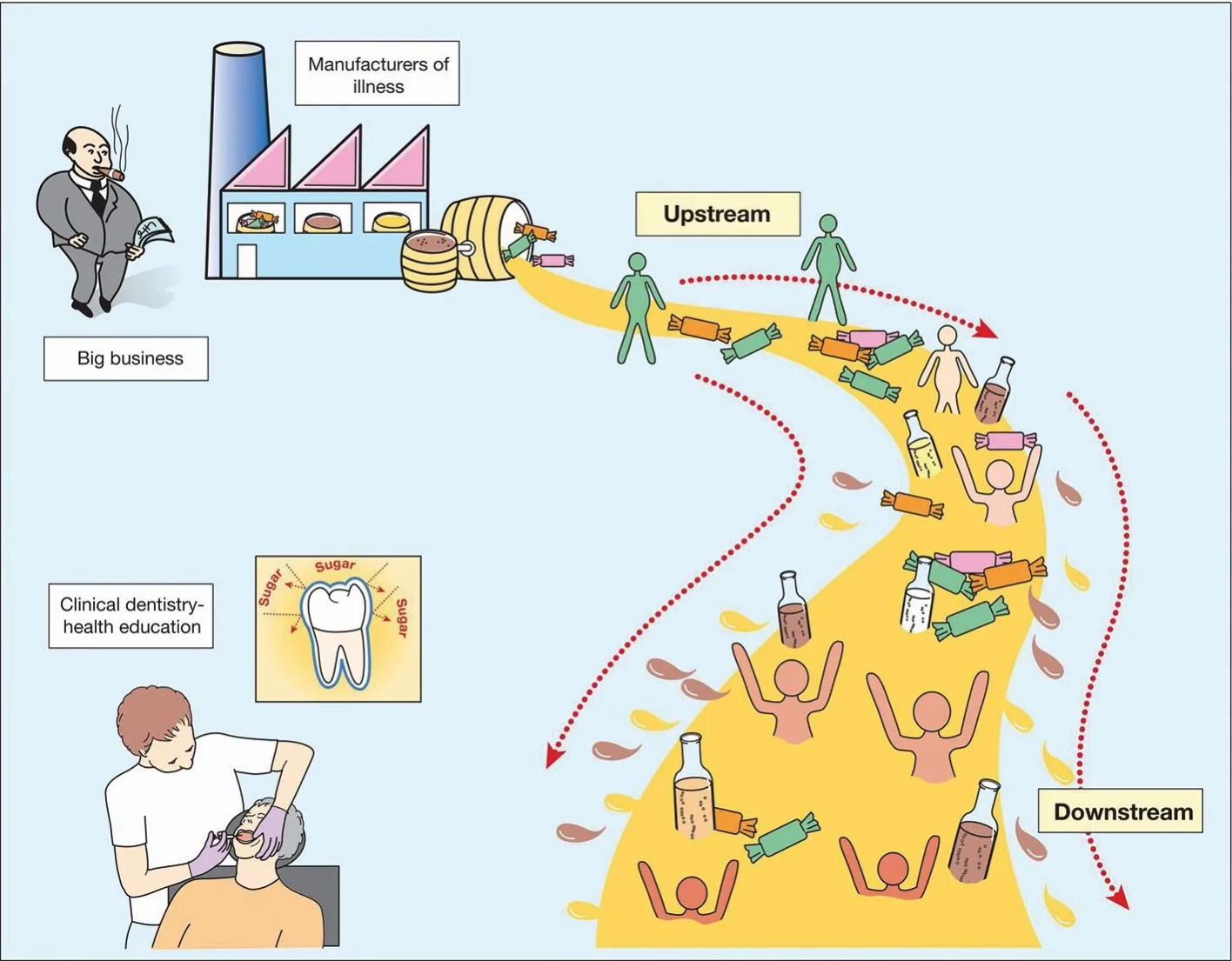
The concept of upstream and downstream in the context of oral health promotion (reproduced from Chestnutt I G, *Dental Public Health at a Glance,* John Wiley & Sons, 2024, with permission)^33^

The concept of downstream and upstream actions (Fig. 1) to improve oral health are well recognised.^10^ Downstream interventions are those largely directed at individuals, intended to engender behaviour change. These are important in the context of clinical practice. Personalised advice from a dental professional is an important aspect of prevention. There is evidence that advice on oral hygiene procedures provided by dental professionals can improve gingival health.^11^ However, actions dependant on individual behaviour change are difficult to sustain in the long term (how many readers of this article have been able to maintain a New Year's resolution indefinitely?) and have limited potential to impact health at a population level.

If substantial improvements in oral health are to be made, then upstream actions are also required as they are likely to have greater impact. These are interventions which change the environments and conditions in which people are exposed to risk factors through action, normally achieved through legislation and interventions at a population level. It is actions at this level that will ultimately offer the greatest possibility of addressing inequalities in oral health and reduce demands on healthcare resources.

## How commercial interests impact health

**Fig. 2:**
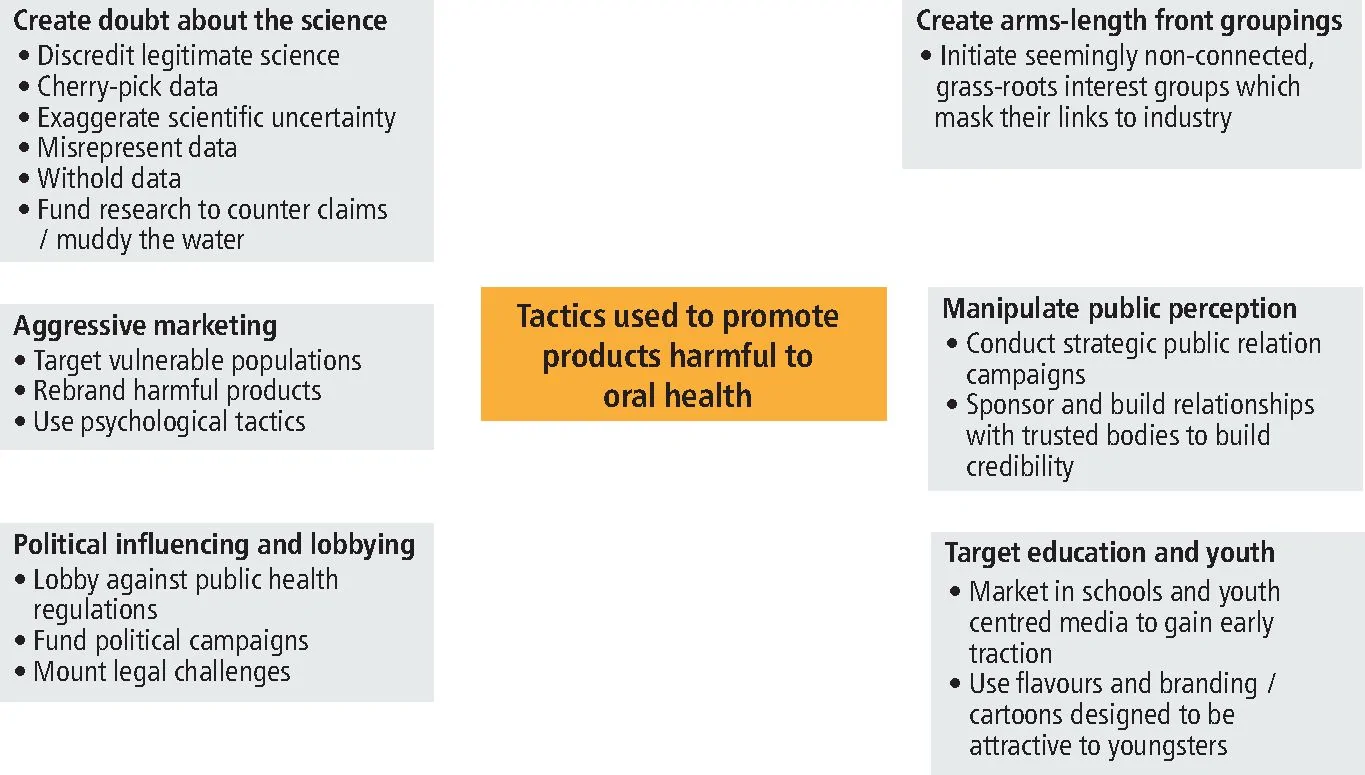
Tactics used to promote products harmful to oral health

The tactics used by commercial actors in marketing products with the potential to adversely affect oral health are illustrated in Figure 2. Over time, these have ranged from deceptive practices to the use of soft power to manipulate public knowledge and perception.

The efforts made by tobacco companies to mask the adverse consequences of using their products are well known.^12^^,^^13^ When regulatory and public health pressures intensified in developed countries, tobacco barons shifted their efforts to the developing world^14^ and have expanded into emerging market areas, such as electronic cigarettes (vapes),^15^ the true health effects of which have yet to be fully established.

Similar strategies have been employed by the sugar industry. Evidence emerged in the last decade of the industry attempting to influence the sugar reduction policies of the World Health Organization.^16^ The sugar industry has also funded research to cast doubt on the effectiveness of sweetened beverages taxation in Mexico.^17^ Sponsorship of sporting events and marketing of products by sports icons are used to associate an image of health, while branding and packaging designed to appeal to children and young people have targeted vulnerable populations.

In dentistry, there is evidence that when the sugar industry could not deny the role of sugar in causing dental caries, it successfully persuaded the National Institute of Dental Research to focus attention to public health interventions that would reduce the harms of sugar consumption, such as fluoride use, rather than reducing sugar intake.^18^ As a result, effort was devoted to researching the role of fluoride as a caries preventive agent, rather than efforts to limit sugar intake.

Dental professionals therefore need to be aware of the subtleties of the tactics used by global corporations as they advertise and market their products.

While this review focuses on the influence of commercial actors with relevance to oral health, there are other commercial forces, such as the gambling and fossil fuel industries, that significantly impact health, both directly and indirectly. However, these lie beyond the scope of this article.

## Options available to government and other agencies with responsibility for promoting and improving health

**Fig. 3:**
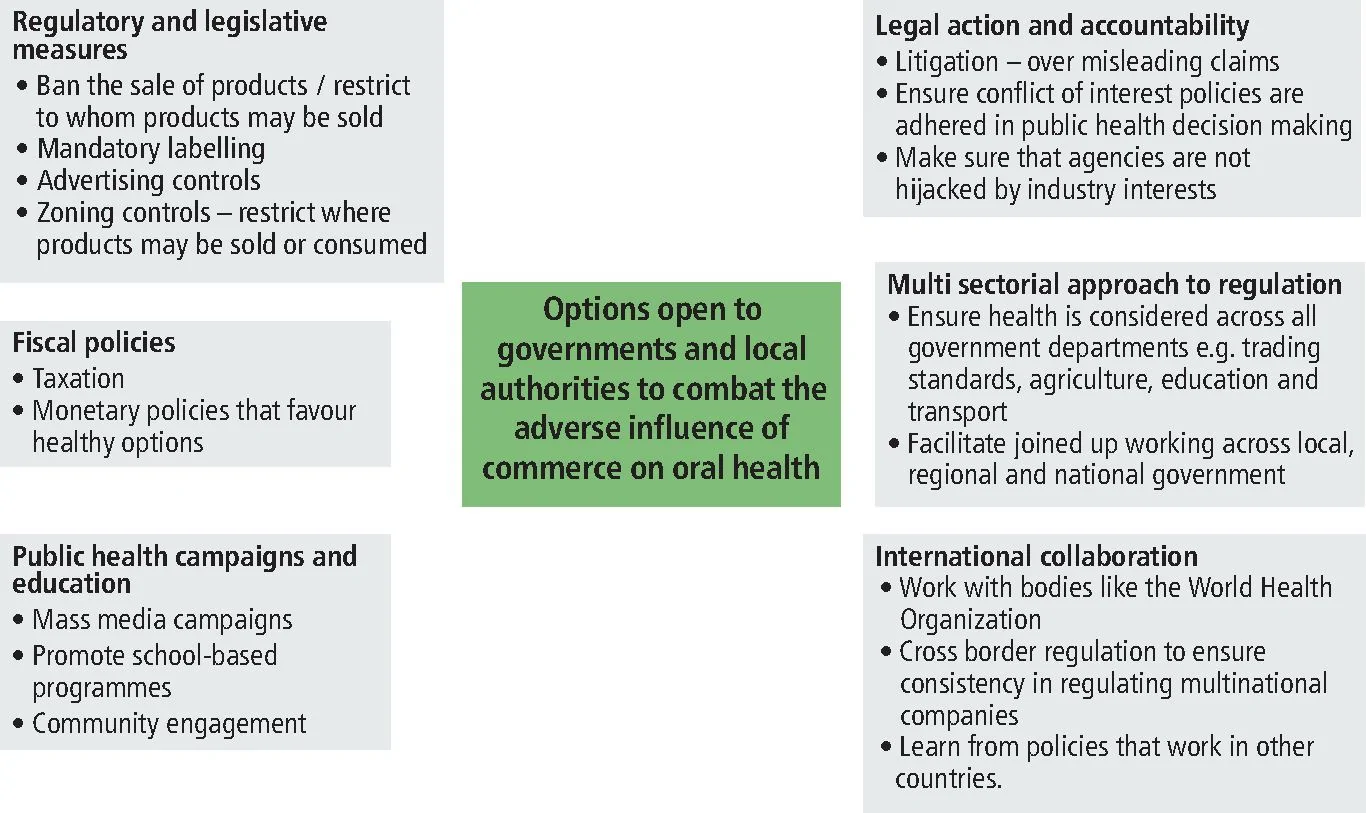
Options open to governments and local authorities to combat the adverse influence of commerce on oral health

Governments and local authorities have at their disposal regulatory and fiscal policies, which can be used to mitigate the health-harming actions of commercial actors (Fig. 3).

This is a complex ethical area that raises the role of the state versus the liberty of the population. Particularly since the global rise of populist politics in the last ten years debates on ‘the nanny state' versus ‘freedom to choose' have dominated the discourse about the use of upstream interventions to achieve health improvement.

### Legislative measures

Outright bans and legally enforceable legislation can be effective. The bans on smoking in public places in the United Kingdom (UK) introduced in the mid-2000s is arguably one of the most significant public health measures of recent decades. Building on this, the UK Government is now advancing a progressive generational ban on the sale of cigarettes through the Tobacco and Vapes Bill, which will make it illegal for people born after 2008 to buy cigarettes.^19^

### Fiscal policy

Fiscal policies, such as taxation, subsidies or public investment, are also effective in controlling commercial actors. The King's Fund have produced an excellent review of the role taxes and regulation play in promoting better health.^20^

Taxes are usually levied by governments to generate income, but in the context of the commercial determinants of health, they have for many years been used as a means of reducing the demand for products harmful to health. The most notable example of where taxation has been used to discourage consumption of harmful products is tobacco. Taxing tobacco products and increasing such taxes at a rate much greater than general price inflation, has long formed a key part of UK tobacco control policies.^21^ As of 2025, the average price of 20 cigarettes in the UK is around £16, approximately 80% of which is tax. A risk in increasing the price of cigarettes is the development of a black market of illegal, fake and smuggled products. It is estimated that in the UK, of all cigarettes smoked every year, 10% (2 billion cigarettes) are illicit.^22^

A recent systematic review^23^ has shown that taxing foods high in fat, salt and sugar is effective in reducing demand, especially if combined with subsidies on healthy foods. Furthermore, Pindea and colleagues'^23^ work suggests that tax effects differed by income level, with the lowest-income groups being most responsive. While this may well contribute to addressing health inequalities, it also raises philosophical questions. Taxes on unhealthy food are often described as regressive, meaning they take up a larger proportion of income of the most disadvantaged. Is it therefore fair to ‘punish' the less well-off with financial penalties to enable them to secure better health?

The most recent fiscal policy of significant impact on oral health has been the UK Soft Drinks Industry Levy (SDIL), more commonly known as the ‘sugar tax'. Imposed on sugar-containing soft drinks in the UK in 2016, this is a two-tier levy applied to drinks with ≥5 g of sugar per 100 ml. His Majesty's Treasury^24^ has recently reported that between 2015 and 2020, there was a 46% average reduction in the sugar content of soft drinks within the scope of the SDIL, and currently 89% of soft drinks sold in the UK are sufficiently low or free from sugar to not qualify for the ‘sugar tax'. This policy approach is held to have been effective in encouraging manufacturers to modify both sugar content and formulation. Clearly this is good news for health in general and from a dental perspective, potentially reduces dental caries risk due to decreased exposure to free sugars. Of course, the SDIL does nothing to address the erosive potential of soft drinks. Sugar-free drinks cause loss of enamel surface to the same degree as sugared drinks.^25^ Milk-based drinks are excluded from the SDIL, despite high levels of free sugars in milkshake-type products. While there is no publicly available evidence that commercial actors were responsible for the exclusion of these products, there are some indications that industry influence may have played a role in shaping the policy. As of 2025, the UK Government is now reviewing the SDIL and is considering whether to remove the exemption for milk-based and milk-substitute drinks, a position supported by the British Dental Association and the British Association for the Study of Community Dentistry, among others.

A further fiscal measure aimed at reducing alcohol consumption, particularly among heavy drinkers and young people, is the minimum unit price (MUP). Both Scotland and Wales have a MUP of 50 pence. This measure has yet to be adopted in England. Evidence from both countries suggests that MUP is effective in reducing the availability and consumption of the cheapest, strongest alcoholic drinks, which tend to be favoured by those at greatest risk of alcohol-related harm.^26^^,^^27^ However, the impact on the alcohol consumption of the general population is less evident. Importantly, MUP is not a tax, unlike the SDIL, with additional revenue from the higher prices going to retailers and producers, not the Scottish or Welsh governments.

### Voluntary agreements

Mandatory legislation is frequently fought or legally challenged by commercial actors. An often-rehearsed argument is that it is better for government and industry to have a voluntary arrangement and those supporting industry often argue for self-regulation. Persuaded by this argument, the Conservative Government in 2011 introduced the Public Health Responsibility Deal.^28^ The idea was that partners signing up to the responsibility deal would use their influence to improve public health. However, a comprehensive review by Knai and colleagues^29^ concluded that the Responsibility Deal did not meet its objectives. Panjwani and Caraher^30^ went further in the criticism of the Public Health Responsibility Deal, writing: ‘The Responsibility Deal approach is fundamentally flawed in its expectation that industry will take voluntary actions that prioritise public health interests above its own. Being government-led counts for little in the absence of sanctions to drive compliance. Instead, the initiative affords private interests the opportunity to influence in their favour the public health policies and strategies that affect their products'.

### Changing behaviours and attitudes

An old mantra in public health is ‘make healthy choices the easy choices'. Governments and local authorities who have the courage to legislate and use their fiscal powers effectively have the opportunity not only to make healthy choices easy, they have the power to eliminate the need for choice – default. Creating healthier public places can involve removing the burden of choice altogether, such as the removal of drinks high in sugars from vending machines in schools, hospital and leisure centres.

Sometimes public support for legislative change for health is greater than might be imagined.^20^ Although opposition arises, this is likely a small, albeit vocal, minority who hold back progress.

A further consideration is that attitudes change with time. In less than a lifetime we have gone from the position where a widely accepted social norm has gone to an increasingly marginalised behaviour. We have moved from a tobacco-saturated society in which smoking cigarettes was an accepted norm, to one where smoking in public places, such as restaurants, would nowadays see the smoker regarded as a pariah. This transformation illustrates how upstream actions can coalesce to reshape behaviours at a population level.

## Commercial actors within the oral healthcare system

### Oral health industry

**Fig. 4:**
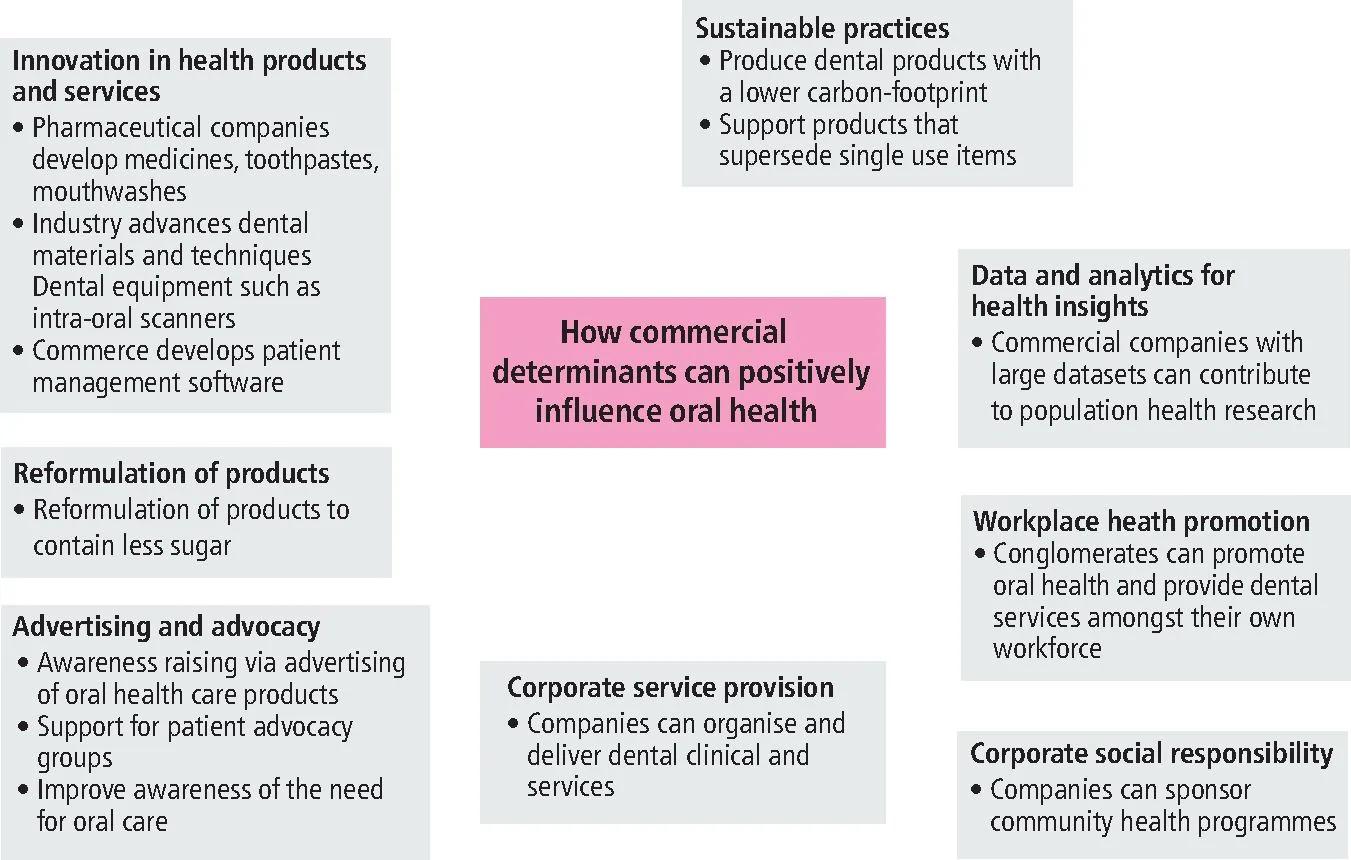
How commercial actors can positively influence oral health

While much of the current interest in the commercial determinants of health in the wider public health field currently concentrate on negative impacts,^7^ it should be remembered that many of the advances in dentistry and oral health have only been possible by the collective efforts of the commercial sector (Fig. 4).

The dramatic improvements seen in oral health in the UK in the latter half of the 20^th^ century are largely attributed to the introduction and widespread use of fluoride-containing toothpaste.^31^ The formulation and testing of these pastes was largely due to the efforts of toothpaste manufacturers. In a similar vein, advertisements for toothpastes and other oral care products are themselves a midstream health intervention, forming a continual reminder to the public of the steps necessary to maintain good oral health.

However, the focus of oral health industry is changing. While oral health products have long been marketed for their dual health and cosmetic benefits, messages increasingly focus on cosmetics. This reflects growing consumer demand but also changes public messaging about the goals of oral health practices. Oral hygiene is being reframed from something that keeps you healthy and free from disease to something that will make you more beautiful and socially desirable.

Beyond consumer products, the infrastructure and products necessary to operate a dental surgery, ranging from the software used to make appointments and document patients' records to the dental chair in which they sit, and the scanners which can now digitally record their dentition, have all been developed by industry.

### Oral healthcare systems

The provision of healthcare involves money, whether directly paid by patients, via an insurance scheme, or a state-funded system such as the National Health Service (NHS). In countries where care is predominantly provided on a private basis, it is well recognised that inequities in access can arise. Often this will lead to those with the greatest need having the least access to care. Even in countries which provide state-funded care, advances in healthcare and technology risk outstripping the money available for dentistry. As a result, state-funded dental care often has to be prioritised or rationed, either explicitly or implicitly.

Reimbursement models, whether state- or insurance-based, can influence not only who is prioritised for care but also the type of care provided. Similarly, fee-for-service models may encourage more profitable, and potentially unnecessary, operative procedures over preventive care.

### Dental professionals

The dental profession can also act as commercial determinants through their influence over public perception of dental need, access to care and the type of treatment provided.

There are well-described difficulties with recruitment of dental professionals in remote and rural locations in the UK.^32^ This can leave these communities with limited access to care.

In the UK, much of what patients want or perceive as necessary for oral health is not available via the NHS. Dental implants and tooth-whitening treatments are two prominent examples. As a result, the dental profession is increasingly navigating two roles – the healthcare provider and the aesthetic industry practitioner. It is not difficult to find dental practices who have prominent deals on their website for such treatments. Indeed, such actors will even facilitate personal loans to facilitate treatment which cannot be afforded as a one-off payment. The ethics of encouraging debt to pay for cosmetic dental treatment is outwith the bounds of this current discussion. What is more pertinent is the inevitable widening of the oral health gap across social class boundaries, when treatments are available only if they can be afforded.

## The role of dental professionals in the wider debate

Dental professionals have a role in advocating for oral health beyond their responsibilities to their individual patients in a clinical setting, and this requires engagement in the conversation about the commercial determinants of health. The factors discussed in this article should provide a foundation of how commercial actors shape general and oral health. Members of the dental team should, when the opportunity arises, support initiatives to curtail the excesses of commerce and push for those in authority to implement laws and fiscal policy that favour health.

## Conclusions

In summary, the three major dental diseases – dental caries, periodontal disease and oral cancer – have sugar, tobacco and alcohol as important causative and risk factors. These are products which are at the heart of the interests of global commercial enterprises. To improve population health and reduce oral health inequalities, action is necessary at both regulatory and fiscal levels to combat the effects of these products.

The oral health industry, dental care systems, and dental professionals are also part of the commercial environment which shape oral health. The way we design, deliver and market services contributes to health outcomes for patients and the public and can widen, or narrow, inequalities.

## Data Availability

All data is available in the accompanying references.
